# Supplementary material for: Feasibility of reporting results of large randomised controlled trials to participants: experience from the Fluoxetine Or Control Under Supervision (FOCUS) trial
Source: BMJ Open. 2020 Nov 30;10(11):e040492. doi: 10.1136/bmjopen-2020-040492 (PMC7705542; doi:10.1136/bmjopen-2020-040492)
Supplement: Supplementary data [file bmjopen-2020-040492supp001.pdf]

E mail subject : FOCUS trial results for participants and their families

Dear Sir/Madam

You are receiving this email because you, or someone close to you, very kindly volunteered to join the FOCUS trial between September 2012 and March 2017. The FOCUS trial was a research study which aimed to find out whether fluoxetine improves recovery after stroke.

We are contacting you now because you, or someone close to you, told us that you would like to know the results of the study and whether you were given fluoxetine or the placebo. You provided this email address as the best method of contact.

We would like to offer our thanks to you for participating in this study

Why we did the FOCUS trial

Strokes can cause weakness of arms and legs, problems with speech, eyesight, swallowing, and in the longer term problems with memory, concentration and fatigue. Although some of these problems improve over time, many people are left with long-term problems after a stroke.

The FOCUS study aimed to find out whether a drug called fluoxetine improves patients' recovery so that they have fewer long-term problems. Fluoxetine is manufactured by several different companies which use their own trade names e.g. Prozac

Fluoxetine has been used for many years to treat people with depression. However, small research studies had suggested that it might also improve recovery after stroke by helping the brain repair itself.

We wanted to find out whether patients given one fluoxetine capsule each day for six months after a stroke recovered better than those given a placebo (or dummy) capsule. The two capsules looked identical, so neither the patients, or the doctors or nurses knew whether the patient was receiving the fluoxetine capsule or the dummy capsule. Altogether, 3127 patients from 103 stroke units from all over the UK took part in the trial.

Your (or someone close to you) treatment allocation

You (or someone close to you) were allocated to take:

< treatment> on <date of randomization>

About a third of patients did not take the allocated treatment for six months, for a wide variety of reasons but often because they developed symptoms which they thought were due to the capsules. Interestingly, the number of patients stopping the treatment early was similar in those taking fluoxetine and those taking the placebo.

What the FOCUS trial has shown

The FOCUS trial showed that fluoxetine made no difference to overall recovery.

However, people who were taking fluoxetine were less likely to become depressed by the follow-up at six months than those taking the placebo (13% taking fluoxetine developed depression compared with 17% taking placebo).

People taking fluoxetine were slightly more likely to break a bone in the first six months - 2·9% of those who were taking fluoxetine fractured a bone compared with 1·5% of those taking placebo.

What effect will this have on future patients?

These results mean that doctors will not now routinely use fluoxetine in the hope that it will improve patients' overall recovery. However, the information on the reduced chance of developing depression and the increased chance of fracturing a bone will help patients, families and their doctors decide whether to take fluoxetine for six months after a stroke.

Do these results have any implications for my current treatment?

No, these results have no direct relevance to your current treatment (or that of someone close to you) since they relate to treatment in the first six months after a stroke, and not treatment in the longer term. If you are taking fluoxetine at the moment then you should not stop, but if you are concerned discuss your treatment with your doctor.

If you would like more information about the FOCUS study click on the links below:

Mead G, Dennis M. *BMJ Open* 2020; 10:e040492. doi: 10.1136/bmjopen-2020-040492

Links to

Original patient information leaflet

Blank consent form

Final Newsletter for participants (a pdf of this email without allocation)

Published paper (link to Lancet paper hopefully)

Study identifiers
Research Ethics Committee Ref: 11/SS/0100
Protocol No: FOCUS12
Eudra CT No: 2011-005616-29
IRAS: 84669

How to contact us if you have any questions

If you would like to ask any questions about the results, or your participation in the study, please don't hesitate to contact us either by email or telephone. The telephone information line will be open until 31st March 2019

Email: focustrial@ed.ac.uk

FOCUS trial information line (9am-5pm Mon-Fri): 0131 242 7741
(if we do not answer immediately please leave a message with the patients name, and date of birth and a telephone number and we will get back to you)

Thank you very much once again for taking part in the FOCUS trial. It is only through the generosity of people like yourself that we can find out how better to treat patients with stroke in the future. We are most grateful to you.

Yours sincerely

Prof Martin Dennis Prof Gillian Mead

Mead G, Dennis M. *BMJ Open* 2020; 10:e040492. doi: 10.1136/bmjopen-2020-040492
