# Supplementary material for: Feasibility of reporting results of large randomised controlled trials to participants: experience from the Fluoxetine Or Control Under Supervision (FOCUS) trial
Source: BMJ Open. 2020 Nov 30;10(11):e040492. doi: 10.1136/bmjopen-2020-040492 (PMC7705542; doi:10.1136/bmjopen-2020-040492)
Supplement: Author's manuscript [file bmjopen-2020-040492.draft_revisions.pdf]

BMJ Open is committed to open peer review. As part of this commitment we make the peer review history of every article we publish publicly available.

When an article is published we post the peer reviewers' comments and the authors' responses online. We also post the versions of the paper that were used during peer review. These are the versions that the peer review comments apply to.

The versions of the paper that follow are the versions that were submitted during the peer review process. They are not the versions of record or the final published versions. They should not be cited or distributed as the published version of this manuscript.

BMJ Open is an open access journal and the full, final, typeset and author-corrected version of record of the manuscript is available on our site with no access controls, subscription charges or pay-per-view fees (http://bmjopen.bmj.com).

If you have any questions on BMJ Open's open peer review process please email info.bmjopen@bmj.com

BMJ Open

Feasibility of reporting results of large randomised controlled trials to participants: experience from the Fluoxetine or Control under supervision (FOCUS) trial

Journal:	*BMJ Open*
Manuscript ID	bmjopen-2020-040492
Article Type:	Original research
Date Submitted by the Author:	18-May-2020
Complete List of Authors:	Mead, Gillian; University of Edinburgh, Geriatric Medicine<br>Dennis, Martin; Neurosciences Trials Unit
Keywords:	STROKE MEDICINE, GERIATRIC MEDICINE, STATISTICS & RESEARCH METHODS

SCHOLARONE™
Manuscripts

I, the Submitting Author has the right to grant and does grant on behalf of all authors of the Work (as defined in the below author licence), an exclusive licence and/or a non-exclusive licence for contributions from authors who are: i) UK Crown employees; ii) where BMJ has agreed a CC-BY licence shall apply, and/or iii) in accordance with the terms applicable for US Federal Government officers or employees acting as part of their official duties; on a worldwide, perpetual, irrevocable, royalty-free basis to BMJ Publishing Group Ltd ("BMJ") its licensees and where the relevant Journal is co-owned by BMJ to the co-owners of the Journal, to publish the Work in this journal and any other BMJ products and to exploit all rights, as set out in our licence.

The Submitting Author accepts and understands that any supply made under these terms is made by BMJ to the Submitting Author unless you are acting as an employee on behalf of your employer or a postgraduate student of an affiliated institution which is paying any applicable article publishing charge ("APC") for Open Access articles. Where the Submitting Author wishes to make the Work available on an Open Access basis (and intends to pay the relevant APC), the terms of reuse of such Open Access shall be governed by a Creative Commons licence – details of these licences and which Creative Commons licence will apply to this Work are set out in our licence referred to above.

Other than as permitted in any relevant BMJ Author's Self Archiving Policies, I confirm this Work has not been accepted for publication elsewhere, is not being considered for publication elsewhere and does not duplicate material already published. I confirm all authors consent to publication of this Work and authorise the granting of this licence.

For peer review only

Title page

Feasibility of reporting results of large randomised controlled trials to participants: experience from the Fluoxetine or Control under supervision (FOCUS) trial

Authors: FOCUS trial collaboration

(writing committee:

Gillian Mead and Martin Dennis, Centre for Clinical Brain Sciences, Chancellor's Building, Little France Crescent, Edinburgh EH16 4SA)

Corresponding author: Professor Gillian Mead, gillian.e.mead@ed.ac.uk

For peer review only

For peer review only

Article summary

Strengths and limitations of this study

- This is the first large trial to report disseminating results to participants, including treatment allocation.
- We demonstrated the feasibility of our approach which was approved by regulatory authorities.
- However, by using email to disseminate results, this meant that some emails were returned unrecognised.

For peer review only

Abstract 268 words

Objectives:

Informing research participants of the results of studies in which they took part is viewed as an ethical imperative. However, there is little guidance in the literature about how to do this. The Fluoxetine or Control Under Supervision Trial (FOCUS) randomised 3127 patients with a recent acute stroke to six months of fluoxetine or placebo and was published in Lancet on 5th December 2018. The trial team decided to inform the participants of the results at exactly the same time as the Lancet publication, and also whether they had been allocated fluoxetine or placebo. In this report, we describe how we informed participants of the results.

Design. In the 6 month and 12 month follow-up questionnaires, we invited participants to provide an email address if they wished to be informed of the results of the trial. We re-opened our trial telephone helpline between 5th December 2018 and 31st March 2019.

Setting: UK Stroke services

Participants: 3127 participants were randomised. 2847 returned 6 month follow-up forms and 2703 returned 12 month follow-up forms; the remaining participants had died (380), withdrawn consent or did not respond.

Results

Of those returning follow-up questionnaires, a total of 1845 email addresses were provided and a further 50 people requested results to be sent by post. Results were sent to all email and postal addresses provided; 309 emails were returned unrecognised. Seventeen people replied, of whom three called the helpline and the rest responded by email.

Conclusion

It is feasible to disseminate results of large trials to research participants, though only around 60% wanted to receive the results. The system we developed was efficient and required very little resource-and could be replicated by trialists in the future.

Introduction

Eighteen years ago, an article published in the Journal of the American Medical Association recommended that participants are informed of the results of the clinical trials in which they participate [1]. In November 2019, an editorial in the British Medical Journal stated that 'The results of clinical trials should be disseminated to those who took part in them because this is courteous and an ethical imperative' [2]. The World Medical Associations Declaration of Helsinki states 'all medical research subjects should be given the opportunity of being informed about the general outcome and results of the study'. The National Institute of Health Research states that it is important to establish whether a participant will want to be actively informed of trial results, or whether they would like the onus to be left with them to obtain the results [3].

Contacting participants many years after enrolment might be an upsetting reminder of their illness, though one small study in cancer suggested that informing participants might increase their understanding of the trial results [4]. Furthermore, some might argue that trying to contact participants who have died might not be ethical. There is, however, no practical guidance in the literature to our knowledge about the steps required to inform participants of the results of the trials in which they participated. Furthermore, it is not known whether research participants do wish to receive results of the trial, which can sometimes be many years after they had been enrolled.

In this brief report, we describe how we disseminated the results of a large multicentre randomised controlled trial: Fluoxetine or Control Under Supervision (FOCUS), and individuals' treatment allocation, and the feedback received from participants.

For peer review only

Methods

FOCUS was a pragmatic, multicentre, parallel group, double-blind, randomised, placebo-controlled trial done at 103 hospitals in the UK and recruiting 3127 patients between Sept 10, 2012, and March 31, 2017, testing whether a 6 month course of fluoxetine given 2-15 days after stroke would improve recovery at 6 months [5]. This trial is registered with the ISRCTN registry, number ISRCTN83290762

Patient and public involvement:

We involved a Patient and Public Involvement (PPI) group of stroke survivors and carers during its design. The group recommended disseminating results to participants who had taken part; this included the family members of participants who had died; the rationale being that family members might be interested in results of the trials in which their loved one had participated. Our grant application to National Institute of Health Research Health Technology Assessment included a request for funding for dissemination of results and this was approved. Scotland A Research Ethics Committee approved the trial, including our plans to inform participants of results.

Subsequently, two lay members identified through the UK Stroke Research Network who had not been involved in the initial planning stages were invited to sit on the trial steering committee, and advised on how to disseminate results to participants.

Methods of trial follow-up

All the trial follow-ups were by postal questionnaires at 6 and 12 months; participants who did not respond were contacted by telephone and the questionnaires completed over the telephone. The postal questionnaire at both 6 months and 12 months concluded with 'If you want to find out more about the trial and its results (in about 2018) please enter an email address where we can contact you, or a person close to you'. We did not record whether the email address provided was for the participant or for someone else (e.g. a family member).

During follow-up telephone calls with participants who had not returned their 6 month or 12 month follow-up questionnaires, we noted that many participants asked us what their treatment allocation had been. We could not provide this information at the time of the call because we had to remain blinded to treatment allocation, but we decided to include individual treatment allocation when we contacted participants with the results of the trial. To the best of our knowledge, this has never been done before.

We considered whether to disseminate the results to all participants by post but this would have required substantial resource (research staff, paper and postage), and if participants had moved house, confidentiality might have been compromised. Thus, we decided to use whatever email had been provided in the follow-up questionnaires, and only use post if there was no email address.

The content of the email to participants was drafted by the trial team and edited by the two PPI members of the Trial Steering Committee. We did not check whether participants were still alive. Our approach was approved by Scotland A Research Ethics Committee.

When we sent the email (at exactly the same time as the Lancet publication on 5th December 2018) we re-opened the trial telephone helpline (Monday to Friday, 9am to 5pm) until 31st March 2019 so that participants receiving the email could contact us if they had any questions. We recorded how many responses were received and the reason for their response. A co-principal investigator (MSD)

For peer review only

then responded to the participant or family member if this seemed appropriate. We did not follow-up emails that were returned as unrecognised by the mail subsystems.

For peer review only

Results

Of the 3127 participants randomised, 2847 returned 6 month follow-up forms and 2703 returned 12 month follow-up forms; the remaining participants had died (380), withdrawn consent or did not respond. Of the returned forms, 1845 email addresses were provided and a further 50 people requested results by post.

Thus, emails containing the results of FOCUS and treatment allocation were sent from the FOCUS email address (focus.trial@ed.ac.uk) to 1845 email addresses and 50 postal addresses.

309 emails were returned as unrecognised by the email subsystems.

Seventeen people (0.9%) (7 participants, 8 family members and two unknown) replied; of these three called the helpline and the rest replied by email. Replies were all received between 5th and 18th December 2019. Seven expressed thanks for letting them know, two asked for advice on how to read the information we had sent them, and the rest reported on their current health status or informed us of the death of the participant. A co-principal investigator (MSD) telephoned three participants back and emailed the rest of respondents to thank them for contacting us, offered condolences to the bereaved relatives or provided further information that had been requested.

For peer review only

Discussion

We have demonstrated that it is feasible to inform trial participants of the results of a large, pragmatic clinical trial and also their treatment allocation. This required planning, involvement of PPI representatives and approval from the Research Ethics Committee, but surprisingly little resource in terms of research staff time and consumables.

However, almost half of participants did not wish to be informed of results. We did not explore the reasons for not wishing to receive the results. After we had disseminated the results, only a handful contacted us, mostly to thank us for letting them know. All the responses were within a few days of receiving the results.

To the best of our knowledge, this is the first large randomised trial to report experiences of informing research participants of the results of the trial and also treatment allocation. We did not try to contact participants by post if the email had been 'bounced'; this was for practical reasons of resources and cost. We did not record whether the email address provided for receipt of results was for the patient or for a family member, and so we cannot report how many results were received by the participants themselves or by a family member.

Are there any ethical problems with informing participants? Our first participants were enrolled in 2012, and the results were sent to them more than six years later. It is possible that participants might have been upset to be reminded of their stroke so long after their enrolment. However, although FOCUS was not designed to explore this, we found no evidence that receiving results was distressing. In theory, families might have been upset to receive an email had their loved one died, but all of the bereaved family members who contacted us expressed their appreciation of having being informed.

Although we have demonstrated that it is feasible to contact research participants by email, we did not formally explore the thoughts and feelings of research participants when they received the results. This would ideally have required a qualitative sub study which was not an aim of our study.

Implications for practice

Disseminating results of trials to research participants should be viewed as an ethical imperative [2]. The model for dissemination that we developed with our PPI representatives was feasible. We recommend that trialists consider using our approach, that funders provide funding for this, and that ethics committees approve future requests to use this approach.

Implications for research

Further research is required to explore why some participants do not wish to receive results of studies in which they participated, whether participants generally wish to know individual treatment allocation, and how they wish to receive the information (e.g. by email, by post, or being provided with a link to a website).

For peer review only

Summary boxes

What is already known on this topic

It is widely agreed that research participants should be informed about the results of research in which they participate.

However, there is no guidance about how to do this.

What this study adds

Our study has shown that it is feasible to collect email addresses at follow-up and inform participants by email of the results, and their individual treatment allocation, at exactly the same time as the study is published in a scientific journal.

Just over half of research participants wished to know the results.

For peer review only

Acknowledgements

The start-up phase of the FOCUS trial was funded by the UK Stroke Association (TSA 2011101) and the main phase funded by the NIHR Health Technology Assessment Programme (project number 13/04/30). The views and opinions expressed herein are those of the authors and do not necessarily reflect those of the NIHR Health Technology Assessment Programme. Recruitment and follow-up was supported by the NIHR-funded UK Stroke Research Network and the Scottish Stroke Research Network, which was supported by NHS Research Scotland (NRS).

Scotland A Research Ethics Committee approved the trial (11/SS/0100) on 21.12.2011.

We would like to thank all the participants and their families, the Dundee Speakeasy group who devised the accessible patient information leaflets, and the PPI group who advised us on the design of FOCUS and advocated for participants to be informed of results. We would like to thank David Burgess (a PPI member of our group) for helpful comments on this manuscript.

References

[1] Partridge AH, Winer EP. Informing Clinical Trial Participants About Study Results. *JAMA.* 2002;288(3):363–365. doi:https://doi.org/10.1001/jama.288.3.363

[2] Taylor J. Reporting research findings to participants. BMJ 2019;367:16324

[3] NIHR website http://www.ct-toolkit.ac.uk/routemap/dissemination-of-results/. Accessed 01/12/2019

[4] Mancini J, Genre D, Dalenc F, Ferrero JM, Kerbrat P, Martin AL, Roché H, Maylevin F, Tarpin C, Viens P, Genève J, Julian-Reynier C. Participants' uptake of clinical trial results: a randomised experiment. *Br J Cancer*. 2010 Mar 30;102(7):1081-4. doi: 10.1038/sj.bjc.6605592. Epub 2010 Mar 2. PMID: 20197767; PMCID: PMC2853089.

[5] FOCUS Trial Collaboration (writing group Martin Dennis (Chair) John Forbes, Catriona Graham, Maree Hackett, Graeme J Hankey, Allan House, Stephanie Lewis, Erik Lundström, Peter Sandercock, Gillian Mead) Effects of fluoxetine on functional outcomes after acute stroke (FOCUS): a pragmatic, double-blind, randomised, controlled trial*. Lancet* Published Online December 5, 2018 http://dx.doi.org/10.1016/ S0140-6736(18)32823-X

Copyright

The Corresponding Author has the right to grant on behalf of all authors and does grant on behalf of all authors, an exclusive licence on a worldwide basis to the BMJ Publishing Group Ltd to permit this article (if accepted) to be published in BMJ editions and any other BMJPGL products and sublicences such use and exploit all subsidiary rights, as set out in the licence.

Competing interests

Both authors have completed the ICMJE uniform disclosure form. The authors declare no support from any organisation for the submitted work; no financial relationships with any organisations that might have an interest in the submitted work in the previous three years, no other relationships or activities that could appear to have influenced the submitted work.

Contribution

For peer review only

Professors Mead and Dennis were co-principal investigators of the FOCUS trial and co-led all aspects of the trial from inception to completion and dissemination.

Guarantor

Professor Mead is the guarantor

Data sharing

Data will be made available upon reasonable request.

Transparency

Professor Mead, the lead author and the manuscript's guarantor, affirms that the manuscript is an honest, accurate, and transparent account of the study being reported; that no important aspects of the study have been omitted; and that any discrepancies from the study as planned (and, if relevant, registered) have been explained.

For peer review only

BMJ Open

Feasibility of reporting results of large randomised controlled trials to participants: experience from the Fluoxetine or Control under supervision (FOCUS) trial

Journal:	*BMJ Open*
Manuscript ID	bmjopen-2020-040492.R1
Article Type:	Original research
Date Submitted by the Author:	18-Aug-2020
Complete List of Authors:	Mead, Gillian; University of Edinburgh, Geriatric Medicine<br>Dennis, Martin; Neurosciences Trials Unit
**Primary Subject Heading**:	Communication
Secondary Subject Heading:	Research methods
Keywords:	STROKE MEDICINE, GERIATRIC MEDICINE, STATISTICS & RESEARCH METHODS

SCHOLARONE™
Manuscripts

I, the Submitting Author has the right to grant and does grant on behalf of all authors of the Work (as defined in the below author licence), an exclusive licence and/or a non-exclusive licence for contributions from authors who are: i) UK Crown employees; ii) where BMJ has agreed a CC-BY licence shall apply, and/or iii) in accordance with the terms applicable for US Federal Government officers or employees acting as part of their official duties; on a worldwide, perpetual, irrevocable, royalty-free basis to BMJ Publishing Group Ltd ("BMJ") its licensees and where the relevant Journal is co-owned by BMJ to the co-owners of the Journal, to publish the Work in this journal and any other BMJ products and to exploit all rights, as set out in our licence.

The Submitting Author accepts and understands that any supply made under these terms is made by BMJ to the Submitting Author unless you are acting as an employee on behalf of your employer or a postgraduate student of an affiliated institution which is paying any applicable article publishing charge ("APC") for Open Access articles. Where the Submitting Author wishes to make the Work available on an Open Access basis (and intends to pay the relevant APC), the terms of reuse of such Open Access shall be governed by a Creative Commons licence – details of these licences and which Creative Commons licence will apply to this Work are set out in our licence referred to above.

Other than as permitted in any relevant BMJ Author's Self Archiving Policies, I confirm this Work has not been accepted for publication elsewhere, is not being considered for publication elsewhere and does not duplicate material already published. I confirm all authors consent to publication of this Work and authorise the granting of this licence.

For peer review only

Title page

Feasibility of reporting results of large randomised controlled trials to participants: experience from the Fluoxetine or Control under supervision (FOCUS) trial

Authors: FOCUS trial collaboration

(writing committee:

Gillian Mead and Martin Dennis, Centre for Clinical Brain Sciences, Chancellor's Building, Little France Crescent, Edinburgh EH16 4SA)

Corresponding author: Professor Gillian Mead, gillian.e.mead@ed.ac.uk

For peer review only

For peer review only

Article summary

Strengths and limitations of this study

- This is the first large trial to report disseminating results to participants, including treatment allocation.
- We demonstrated the feasibility of our approach which was approved by regulatory authorities.
- However, by using email to disseminate results, this meant that some emails were returned unrecognised.

Abstract 285 words

Objectives:

Informing research participants of the results of studies in which they took part is viewed as an ethical imperative. However, there is little guidance in the literature about how to do this. The Fluoxetine or Control Under Supervision Trial (FOCUS) randomised 3127 patients with a recent acute stroke to six months of fluoxetine or placebo and was published in Lancet on 5th December 2018. The trial team decided to inform the participants of the results at exactly the same time as the Lancet publication, and also whether they had been allocated fluoxetine or placebo. In this report, we describe how we informed participants of the results.

Design. In the 6 month and 12 month follow-up questionnaires, we invited participants to provide an email address if they wished to be informed of the results of the trial. We re-opened our trial telephone helpline between 5th December 2018 and 31st March 2019.

Setting: UK Stroke services

Participants: 3127 participants were randomised. 2847 returned 6 month follow-up forms and 2703 returned 12 month follow-up forms; the remaining participants had died (380), withdrawn consent or did not respond.

Results

Of those returning follow-up questionnaires, a total of 1845 email addresses were provided and a further 50 people requested results to be sent by post. Results were sent to all email and postal addresses provided; 309 emails were returned unrecognised. Seventeen people replied, of whom three called the helpline and the rest responded by email.

Conclusion

It is feasible to disseminate results of large trials to research participants, though only around 60% of those randomised wanted to receive the results. The system we developed was efficient and required very little resource-and could be replicated by trialists in the future.

For peer review only

Introduction

Eighteen years ago, an article published in the Journal of the American Medical Association recommended that participants are informed of the results of the clinical trials in which they participate [1]. In November 2019, an editorial in the British Medical Journal stated that 'The results of clinical trials should be disseminated to those who took part in them because this is courteous and an ethical imperative' [2]. The World Medical Associations Declaration of Helsinki states 'all medical research subjects should be given the opportunity of being informed about the general outcome and results of the study'. The National Institute of Health Research states that it is important to establish whether a participant will want to be actively informed of trial results, or whether they would like the onus to be left with them to obtain the results [3].

Contacting participants many years after enrolment might be an upsetting reminder of their illness, though one small study in cancer suggested that informing participants might increase their understanding of the trial results [4]. Furthermore, some might argue that trying to contact participants who have died might not be ethical. There is, however, no practical guidance in the literature to our knowledge about the steps required to inform participants of the results of the trials in which they participated. Furthermore, it is not known whether research participants do wish to receive results of the trial, which can sometimes be many years after they had been enrolled.

In this brief report, we describe how we disseminated the results of a large multicentre randomised controlled trial: Fluoxetine or Control Under Supervision (FOCUS), and individuals' treatment allocation, and the feedback received from participants.

For peer review only

Methods

FOCUS was a pragmatic, multicentre, parallel group, double-blind, randomised, placebo-controlled trial done at 103 hospitals in the UK and recruiting 3127 patients between Sept 10, 2012, and March 31, 2017, testing whether a 6 month course of fluoxetine given 2-15 days after stroke would improve recovery at 6 months [5]. This trial is registered with the ISRCTN registry, number ISRCTN83290762

Patient and public involvement:

We involved a Patient and Public Involvement (PPI) group of stroke survivors and carers during its design. The group recommended disseminating results to participants who had taken part; this included the family members of participants who had died; the rationale being that family members might be interested in results of the trials in which their loved one had participated. Our grant application to National Institute of Health Research Health Technology Assessment included a request for funding for dissemination of results and this was approved. Scotland A Research Ethics Committee approved the trial, including our plans to inform participants of results.

Subsequently, two lay members identified through the UK Stroke Research Network who had not been involved in the initial planning stages were invited to sit on the trial steering committee, and advised on how to disseminate results to participants.

Methods of trial follow-up

All the trial follow-ups were by postal questionnaires at 6 and 12 months; participants who did not respond were contacted by telephone and the questionnaires completed over the telephone. The postal questionnaire at both 6 months and 12 months concluded with ‘If you want to find out more about the trial and its results (in about 2018) please enter an email address where we can contact you, or a person close to you’. We did not record whether the email address provided was for the participant or for someone else (e.g. a family member). We did not collect the email address at the time of recruitment.

During follow-up telephone calls with participants who had not returned their 6 month or 12 month follow-up questionnaires, we noted that many participants asked us what their treatment allocation had been. We could not provide this information at the time of the call because we had to remain blinded to treatment allocation, but we decided to include individual treatment allocation when we contacted participants with the results of the trial. To the best of our knowledge, this has never been done before.

We considered whether to disseminate the results to all participants by post; although we had costed for the postage in the grant application (£1 per letter), we realised that this would have required substantial resource including research staff time (estimated 5 minutes per letter, which is about 125 hours for 1500 letters, plus paper, as well as the postage), and if participants had moved house, confidentiality might have been compromised. Thus, we decided to use whatever email had been provided in the follow-up questionnaires, and only use post if there was no email address.

The content of the email to participants was drafted by the trial team and edited by the two PPI members of the Trial Steering Committee. The email was written in lay language, started by an explanation of why we were contacting them, thanking them again for having taken part, provided their treatment allocation and the overall results of the trial (see supplementary materials), links to further information, and the number for the telephone helpline. We did not check whether participants were still alive. Our approach was approved by Scotland A Research Ethics Committee.

For peer review only

We sent the email at exactly the same time as the Lancet publication on 5th December 2018 and the presentation of the main results at the UK Stroke Forum. We managed to coordinate the emails, the presentation and the publication through close liaison with the Lancet and the UK Stroke Forum Scientific Committee. At the same time, we also re-opened the trial telephone helpline (Monday to Friday, 9am to 5pm) until 31st March 2019 so that participants receiving the email could contact us if they had any questions. We recorded how many responses were received and the reason for their response. A co-principal investigator (MSD) then responded to the participant or family member if this seemed appropriate. We did not follow-up emails that were returned as unrecognised by the mail subsystems.

For peer review only

Results

Of the 3127 participants randomised, 2847 returned 6 month follow-up forms and 2703 returned 12 month follow-up forms; the remaining participants had died (380), withdrawn consent or did not respond. Of the returned forms, 1845 email addresses were provided and a further 50 people requested results by post. Thus, the number who wished to be informed of results is 1895; this represents 60% of all participants and 70% of those who could be contacted at 12 months.

We therefore sent emails containing the results of FOCUS and treatment allocation from the FOCUS email address (focus.trial@ed.ac.uk) to 1845 email addresses, and a paper letter to the 50 postal addresses.

309 emails were returned as unrecognised by the email subsystems.

Seventeen people (0.9%) (7 participants, 8 family members and two unknown) replied; of these three called the helpline and the rest replied by email. Replies were all received between 5th and 18th December 2019. Seven expressed thanks for letting them know, two asked for advice on how to read the information we had sent them, and the rest reported on their current health status or informed us of the death of the participant. A co-principal investigator (MSD) telephoned three participants back and emailed the rest of respondents to thank them for contacting us, offered condolences to the bereaved relatives or provided further information that had been requested.

For peer review only

Discussion

We have demonstrated that it is feasible to inform trial participants of the results of a large, pragmatic clinical trial and also their treatment allocation. This required planning, involvement of PPI representatives, coordination of the email dissemination with the publication of the trial in Lancet, and approval from the Research Ethics Committee, but surprisingly little resource in terms of research staff time and consumables.

However, almost half of participants did not wish to be informed of results. We did not explore the reasons for not wishing to receive the results. After we had disseminated the results, only a handful contacted us, mostly to thank us for letting them know. All the responses were within a few days of receiving the results. Only three people called the helpline-this is far lower than the number who typically contacted the hotline during the trial, which had been about four per week. For future trials, we would probably not reopen the helpline and just provide an email contact address for any queries.

To the best of our knowledge, this is the first large randomised trial to report experiences of informing research participants of the results of the trial and also each person's treatment allocation. We did not try to contact participants by post if the email had been 'bounced'; this was for practical reasons of resources and cost. We did not record whether the email address provided for receipt of results was for the patient or for a family member, and so we cannot report how many results were received by the participants themselves or by a family member.

Are there any ethical problems with informing participants? Our first participants were enrolled in 2012, and the results were sent to them more than six years later. It is possible that participants might have been upset to be reminded of their stroke so long after their enrolment. However, although FOCUS was not designed to explore this, we found no evidence that receiving results was distressing. In theory, families might have been upset to receive an email had their loved one died, but all of the bereaved family members who contacted us expressed their appreciation of having being informed. In a review of empirical research about informing participants, the drawbacks might be increased anxiety, anger, guilt, or upset, whilst benefits might include pleasure, satisfaction, and relief [6].

Although we have demonstrated that it is feasible to contact research participants by email, we did not formally explore the thoughts and feelings of research participants when they received the results. This would ideally have required a qualitative sub study which was not an aim of our study.

<u>Implications for practice</u>

Disseminating results of trials to research participants should be viewed as an ethical imperative [2]. The model for dissemination that we developed with our PPI representatives was feasible. We recommend that trialists consider using our approach, that funders provide funding for this, and that ethics committees approve future requests to use this approach.

<u>Implications for research</u>

Further research is required to explore why some participants do not wish to receive results of studies in which they participated, whether participants generally wish to know individual treatment allocation, and how they wish to receive the information (e.g. by email, by post, or being provided with a link to a website).

For peer review only

For peer review only

Funding

This work was supported by Stroke Association (grant number TSA 2011101) and NIHR Health Technology Assessment Programme (project number 13/04/30). The views and opinions expressed herein are those of the authors and do not necessarily reflect those of the NIHR Health Technology Assessment Programme.

Acknowledgements

Recruitment and follow-up was supported by the NIHR-funded UK Stroke Research Network and the Scottish Stroke Research Network, which was supported by NHS Research Scotland (NRS).Scotland A

Research Ethics Committee approved the trial (11/SS/0100) on 21.12.2011.

We would like to thank all the participants and their families, the Dundee Speakeasy group who devised the accessible patient information leaflets, and the PPI group who advised us on the design of FOCUS and advocated for participants to be informed of results. We would like to thank David Burgess (a PPI member of our group) for helpful comments on this manuscript.

References

[1] Partridge AH, Winer EP. Informing Clinical Trial Participants About Study Results. *JAMA.* 2002;288(3):363–365. doi:https://doi.org/10.1001/jama.288.3.363

[2] Taylor J. Reporting research findings to participants. BMJ 2019;367:16324

[3] NIHR website http://www.ct-toolkit.ac.uk/routemap/dissemination-of-results/. Accessed 01/12/2019

[4] Mancini J, Genre D, Dalenc F, Ferrero JM, Kerbrat P, Martin AL, Roché H, Maylevin F, Tarpin C, Viens P, Genève J, Julian-Reynier C. Participants' uptake of clinical trial results: a randomised experiment. *Br J Cancer*. 2010 Mar 30;102(7):1081-4. doi: 10.1038/sj.bjc.6605592. Epub 2010 Mar 2. PMID: 20197767; PMCID: PMC2853089.

[5] FOCUS Trial Collaboration (writing group Martin Dennis (Chair) John Forbes, Catriona Graham, Maree Hackett, Graeme J Hankey, Allan House, Stephanie Lewis, Erik Lundström, Peter Sandercock, Gillian Mead) Effects of fluoxetine on functional outcomes after acute stroke (FOCUS): a pragmatic, double-blind, randomised, controlled trial. *Lancet* Published Online December 5, 2018 http://dx.doi.org/10.1016/ S0140-6736(18)32823-X

[6] Shalowitz DI, Miller FG, Communicating the Results of Clinical Research to Participants: Attitudes, Practices, and Future Directions, Plos Med 2008; May 13. https://doi.org/10.1371/journal.pmed.0050091

Copyright

The Corresponding Author has the right to grant on behalf of all authors and does grant on behalf of all authors, an exclusive licence on a worldwide basis to the BMJ Publishing Group Ltd to permit this article (if accepted) to be published in BMJ editions and any other BMJPGL products and sublicences such use and exploit all subsidiary rights, as set out in the licence.

Competing interests

Both authors have completed the ICMJE uniform disclosure form. The authors declare no support from any organisation for the submitted work; no financial relationships with any organisations that might have an interest in the submitted work in the previous three years, no other relationships or activities that could appear to have influenced the submitted work.

Contribution

Professors Mead and Dennis were co-principal investigators of the FOCUS trial and co-led all aspects of the trial from inception to completion and dissemination.

Guarantor

Professor Mead is the guarantor

Data sharing

Data will be made available upon reasonable request.

Transparency

Professor Mead, the lead author and the manuscript's guarantor, affirms that the manuscript is an honest, accurate, and transparent account of the study being reported; that no important aspects of the study have been omitted; and that any discrepancies from the study as planned (and, if relevant, registered) have been explained.

For peer review only

E mail subject : FOCUS trial results for participants and their families

Dear Sir/Madam

You are receiving this email because you, or someone close to you, very kindly volunteered to join the FOCUS trial between September 2012 and March 2017. The FOCUS trial was a research study which aimed to find out whether fluoxetine improves recovery after stroke.

We are contacting you now because you, or someone close to you, told us that you would like to know the results of the study and whether you were given fluoxetine or the placebo. You provided this email address as the best method of contact.

We would like to offer our thanks to you for participating in this study

Why we did the FOCUS trial

Strokes can cause weakness of arms and legs, problems with speech, eyesight, swallowing, and in the longer term problems with memory, concentration and fatigue. Although some of these problems improve over time, many people are left with long-term problems after a stroke.

The FOCUS study aimed to find out whether a drug called fluoxetine improves patients' recovery so that they have fewer long-term problems. Fluoxetine is manufactured by several different companies which use their own trade names e.g. Prozac

Fluoxetine has been used for many years to treat people with depression. However, small research studies had suggested that it might also improve recovery after stroke by helping the brain repair itself.

We wanted to find out whether patients given one fluoxetine capsule each day for six months after a stroke recovered better than those given a placebo (or dummy) capsule. The two capsules looked identical, so neither the patients, or the doctors or nurses knew whether the patient was receiving the fluoxetine capsule or the dummy capsule. Altogether, 3127 patients from 103 stroke units from all over the UK took part in the trial.

Your (or someone close to you) treatment allocation

For peer review only - http://bmjopen.bmj.com/site/about/guidelines.xhtml

You (or someone close to you) were allocated to take:

< treatment> on <date of randomization>

About a third of patients did not take the allocated treatment for six months, for a wide variety of reasons but often because they developed symptoms which they thought were due to the capsules. Interestingly, the number of patients stopping the treatment early was similar in those taking fluoxetine and those taking the placebo.

For peer review only

What the FOCUS trial has shown

The FOCUS trial showed that fluoxetine made no difference to overall recovery.

However, people who were taking fluoxetine were less likely to become depressed by the follow-up at six months than those taking the placebo (13% taking fluoxetine developed depression compared with 17% taking placebo).

People taking fluoxetine were slightly more likely to break a bone in the first six months - 2·9% of those who were taking fluoxetine fractured a bone compared with 1·5% of those taking placebo.

What effect will this have on future patients?

These results mean that doctors will not now routinely use fluoxetine in the hope that it will improve patients' overall recovery. However, the information on the reduced chance of developing depression and the increased chance of fracturing a bone will help patients, families and their doctors decide whether to take fluoxetine for six months after a stroke.

Do these results have any implications for my current treatment?

No, these results have no direct relevance to your current treatment (or that of someone close to you) since they relate to treatment in the first six months after a stroke, and not treatment in the longer term. If you are taking fluoxetine at the moment then you should not stop, but if you are concerned discuss your treatment with your doctor.

If you would like more information about the FOCUS study click on the links below:

For peer review only - http://bmjopen.bmj.com/site/about/guidelines.xhtml

Links to

Original patient information leaflet

Blank consent form

Final Newsletter for participants (a pdf of this email without allocation)

Published paper (link to Lancet paper hopefully)

Study identifiers
Research Ethics Committee Ref: 11/SS/0100
Protocol No: FOCUS12
Eudra CT No: 2011-005616-29
IRAS: 84669

How to contact us if you have any questions

If you would like to ask any questions about the results, or your participation in the study, please don't hesitate to contact us either by email or telephone. The telephone information line will be open until 31st March 2019

Email: focustrial@ed.ac.uk

FOCUS trial information line (9am-5pm Mon-Fri): 0131 242 7741
(if we do not answer immediately please leave a message with the patients name, and date of birth and a telephone number and we will get back to you)

Thank you very much once again for taking part in the FOCUS trial. It is only through the generosity of people like yourself that we can find out how better to treat patients with stroke in the future. We are most grateful to you.

Yours sincerely

Prof Martin Dennis   Prof Gillian Mead

For peer review only - http://bmjopen.bmj.com/site/about/guidelines.xhtml
